# Screening gender minority people for harmful alcohol use

**DOI:** 10.1371/journal.pone.0231022

**Published:** 2020-04-07

**Authors:** Annesa Flentje, Branden T. Barger, Matthew R. Capriotti, Micah E. Lubensky, Matthew Tierney, Juno Obedin-Maliver, Mitchell R. Lunn

**Affiliations:** 1 Department of Community Health Systems, School of Nursing, University of California, San Francisco, California, United States of America; 2 Alliance Health Project, Department of Psychiatry, School of Medicine, University of California, San Francisco, California, United States of America; 3 The PRIDE Study/PRIDEnet, School of Medicine, Stanford University, Stanford, California, United States of America; 4 Office of Diversity and Outreach, University of California, San Francisco, California, United States of America; 5 Department of Psychology, San Jose State University, San Jose, California, United States of America; 6 Department of Obstetrics and Gynecology, School of Medicine, Stanford University, Stanford, California, United States of America; 7 Division of Nephrology, Department of Medicine, School of Medicine, Stanford University, Stanford, California, United States of America; Monash University, AUSTRALIA

## Abstract

This study identifies how to screen for harmful alcohol use among gender minority (*e*.*g*., transgender and gender-expansive) people using brief screening methods and identifies which screening methods perform best among gender-expansive, transfeminine, and transmasculine subgroups, as screening recommendations are not currently available. Using 2018 Annual Questionnaire data from The PRIDE Study, area under the curve (AUC) values were compared to identify which screening methods (“4 or more” or “5 or more” drinks on one occasion in the past year, or one or more items from the Alcohol Use Disorders Identification Test [AUDIT]) best predicted (i) harmful alcohol use and (ii) one or more past year alcohol dependence symptoms or consequences. Among 1892 participants, “5 or more” drinks on one occasion (AUC ranges: 0.82–0.86) performed better than “4 or more” drinks (AUC ranges: 0.78–0.81) in predicting harmful drinking. The screening methods “4 or more” drinks, “5 or more” drinks, and the consumption items of the AUDIT (AUDIT-C) using a cutoff score of 3 all maximized sensitivity and specificity to predict alcohol dependence symptoms or consequences in gender minority people overall (AUC: 0.77–0.78). Screening for “5 or more” drinks on one occasion within the past year performed as well as or better than other screening methods to detect both harmful drinking and alcohol dependence-related symptoms or consequences. This single-item screening method can identify if more extensive alcohol use assessment is warranted with gender minority people.

## Introduction

Excessive alcohol use costs the United States over $200 billion annually. The Centers for Disease Control and Prevention indicate that most of these costs can be attributed to binge drinking, defined as “4 or more” drinks per occasion for women or “5 or more” drinks per occasion for men [1]. Excessive alcohol use is related to mortality, impairment at or lost time from work, criminal justice involvement, and health care utilization [1]. Gender minority people, who include transgender individuals (*i*.*e*. individuals who identify with a gender on a masculine or feminine spectrum that does not align with what is commonly associated with the sex assigned to them at birth) as well as gender-expansive individuals (*i*.*e*., individuals who have a gender identity that does not align with a feminine or masculine binary, meaning not limited to man or woman), have higher rates of alcohol use [2,3] than cisgender people (*i*.*e*., people whose gender is the same as the sex assigned to them at birth, for definitions relevant to gender, gender minority people, and sexual minority people see S1 Table). This increased risk has been attributed to minority stress, including experiences of discrimination in society [4].

Screening for harmful alcohol use helps health care providers offer information, brief interventions, and/or provide or refer to treatment, which can reduce alcohol use [5]. A brief screening method is important to increase the feasibility of implementing screening into health care visits, as providers may be more likely to deliver briefer screening methods [6]. The use of a single-item screening method has been recommended by the National Institute on Alcohol Abuse and Alcoholism (NIAAA), which asks, on how many occasions in the past year men have had “5 or more” drinks in one day or women have had “4 or more” drinks in one day [7]. A response of one or more days is indicative of a positive screen for at-risk drinking [7]. This NIAAA-recommended, single-item method has comparable sensitivity and specificity to longer screening instruments in health care settings [8]. The different screening cutoff values used in the NIAAA recommended single-item method for men versus women (“5 or more” versus “4 or more” drinks) were based upon similar rates of alcohol-related problems for men and women at these cutoff values [9], presumably as it relates to underlying differences in alcohol metabolism [10], and have been widely adopted. Brief screening methods such as the 3-item version of the Alcohol Use Disorders Identification Test (AUDIT) known as the AUDIT-C, which focuses on alcohol consumption have high sensitivity and specificity in predicting alcohol use disorders [11], but also has cutoff scores that are different for (presumed cisgender) men and women [12] with no guidance about which cutoff scores to use with gender minority people. A recent systematic review of alcohol research with gender minority populations concludes that research on the use of alcohol use screening methods for gender minority people is urgently needed [13]. Health care providers have no guidance about whether to use screening guidelines associated with their patient’s current gender or the sex assigned to their patient at birth, and no guidance about how to screen for harmful alcohol use among patients who are gender-expansive.

The purpose of this study was to identify how to screen for harmful alcohol use among gender minority people and to identify which screening methods should be used for subpopulations of gender minority people, including gender-expansive, transfeminine, and transmasculine people. We examined whether using a screening method of “4 or more” or “5 or more” alcoholic drinks on one occasion within the past year best identifies gender minority individuals with likely harmful alcohol use as measured by the 10-item AUDIT. We also sought to identify which of the following screening methods pertaining to past year alcohol use best predicted past year experience of alcohol-related dependence symptoms or consequences: “4 or more” drinks on one occasion, “5 or more” drinks on one occasion, the frequency of drinking, the typical number of alcoholic drinks, or the 3-item AUDIT-C (using both cutoff scores previously designated for men and women). We undertook analyses examining these screening methods among gender minority people considered together as a group as well as between and among three gender minority subgroups: gender-expansive, transfeminine, and transmasculine individuals. This study informs brief screening practices for harmful alcohol use among gender minority people in health care settings.

## Methods

Data for this study were collected through The PRIDE Study, a national, prospective, online, longitudinal study of sexual and gender minority people (14). Inclusion criteria were (i) identifying as a sexual and/or gender minority person, (ii) being at least 18 years old, (iii) living in the United States or its territories, and (iv) reading and writing in English. Data came from The PRIDE Study’s 2018 Annual Questionnaire, an annual assessment of overall health [14], and were collected between June 2018 and May 2019 and study was approved by the Institutional Review Boards of the University of California and Stanford University. Participants were included if they reported a gender that differed from the sex assigned to them at birth and completed items related to alcohol use.

### AUDIT

All participants were administered the AUDIT, a 10-item measure that screens for harmful alcohol use [15], where items answered on a scale of 0–4 are summed to obtain a score (range 0–40). The AUDIT consists of 3 items that query alcohol consumption [11], with remaining items related to alcohol dependence and consequences [16]. Consistent with AUDIT instructions, items 2–10 were only administered if participants responded affirmatively to AUDIT item 1, indicating that they had a drink containing alcohol in the past year. Individuals who reported that they had no alcoholic drinks in the last year were coded 0 on subsequent AUDIT items.

### Potential screening methods

#### NIAAA recommended single-item screening method

Based on the NIAAA-recommended screening method, we queried: “How long has it been since you last had [4 or 5] or more drinks containing alcohol on one occasion?” All participants were presented with questions querying “4 or more” and “5 or more”. Items were presented with an NIAAA-produced image depicting standard drinks in hard liquor, beer, and wine [17].

#### Single AUDIT items reflecting quantity and frequency

AUDIT item 1 on the frequency of drinking (“How often do you have a drink containing alcohol?”) and AUDIT item 2 on typical amount of drinking (“How many drinks containing alcohol do you have on a typical day when you are drinking?”) were considered as single-item screening methods.

#### AUDIT-C

AUDIT-C scores were calculated by summing AUDIT consumption items 1, 2, and 3, for a total score of 0–12. We tested both standard AUDIT-C screening cutoff scores of ≥ 3 (typically for women) and ≥ 4 (typically for men) [12].

### Predicted outcomes

#### Harmful alcohol use

Harmful alcohol use was defined as a score ≥ 8 on the AUDIT, consistent with AUDIT screening guidelines [15]. This outcome was tested with potential screening methods of “4 or more” or “5 or more” drinks within the past year, as the other screening methods represented a subset of the AUDIT items represented within this total score.

#### Alcohol dependence symptoms and consequences

Alcohol dependence symptoms and consequences were defined by any affirmative response to AUDIT items 4–9, which query the following experiences within the past year: the inability to stop drinking after starting (item 4), failing to do what is expected due to drinking (item 5), needing to drink in order to get yourself going after a heavy drinking session (item 6), feeling guilt or remorse following drinking (item 7), the inability to remember events that happened while drinking (item 8), and getting injured or injuring another due to drinking (item 9). These items were previously defined as alcohol dependence symptoms and consequences by a factor analysis of the AUDIT [16]. These items were used as an outcome here based on previous research, which used a similar method to develop a screening method for at-risk drinking in a different population, though in that study they referred to these items as “problems due to drinking” [18]. Within this study, we refer to these items as “alcohol dependence symptoms and consequences” based on the previously described factor structure of the AUDIT [16]. This outcome was tested with all potential screening methods.

### Gender minority groups based on gender identity

Participants were divided into three mutually exclusive analysis groups: gender-expansive, transfeminine, or transmasculine based on participant sex assigned at birth and current gender identity. Participants could select one or more gender identities from the following options: (1) Genderqueer, (2) Man, (3) Transgender Man, (4) Transgender Woman, (5) Woman, and (6) Another Gender Identity, for which participants could write in a gender identity. Response options for sex assigned at birth included (1) Female and (2) Male. Participants who reported a non-binary gender identity (*e*.*g*., genderqueer, nonbinary, agender) or more than one gender identity where both were not reflective of the same binary gender (*e*.*g*., man and woman) were categorized into the gender-expansive category. Participants were categorized as transfeminine if they reported a gender/genders that reflected a feminine binary (*e*.*g*., girl, woman, transgender woman) and endorsed male sex assigned at birth. Participants were categorized as transmasculine if they endorsed a gender/genders that reflected a masculine binary (*e*.*g*., boy, man, transgender man) and a female sex assigned at birth.

### Participant characteristics

Additional participant characteristics included race, ethnicity, age, individual annual income, highest level of education attained, and sexual orientation.

### Data analyses

Analyses were performed using Stata/SE 15.1 [19]. Demographic differences and differences on alcohol screening methods and outcomes were examined between gender-expansive, transfeminine, and transmasculine groups using Chi-square or Fisher’s exact (when expected cell counts were <5) and one-way analysis of variance. For each potential screening method and predicted outcome, the area under the curve (AUC) of the receiver operating characteristic (ROC) was calculated. Stata package *roccomp* [20] was used to compare AUC values between the different screening methods to determine which best predicted the outcomes. We ran these analyses for all gender minority people as a combined group first, then separately for gender-expansive, transfeminine, and transmasculine groups.

For each outcome, all potential screening method AUC values were compared to each other. If there were differences in AUC values between the screening methods, the two screening methods with the greatest AUC values (or AUC values that were within 0.02 of each other, representing a potential “tie”) were compared to identify detectable AUC differences (*p <* .05) of the screening methods in predicting the outcome. Since single AUDIT items (1 and 2) offer 5-point response scales, we first identified the cutoff value for these items with the highest AUC for each group (all gender minority people combined, transmasculine only, transfeminine only, and gender-expansive only) before comparing these items to the other screening methods. Finally, the candidate screening methods identified within each of the subgroups of gender minority people were compared between subgroups of gender minority people to identify differences in the AUC values of the screening methods between the subgroups.

## Results

### Participants

Participant characteristics are described in Table 1. Most (65.4%) of the gender minority participants (n = 1,239) were gender-expansive, 22.2% (n = 421) were transmasculine, and 12.3% (n = 232) were transfeminine. There were differences in age (*p* < .001); transfeminine individuals were older (median age 38.0 years), and gender-expansive (median age 27.2 years) and transmasculine (median age 28.2 years) individuals were younger. Among gender-expansive individuals, 86.6% reported a female sex assigned to them at birth. Over half (51.0%, n = 925) of the participants reported making $20,000 or less per year. Participants predominantly identified their ethno-racial background as non-Hispanic/Latino White (81.3%). Most individuals (56.0%, n = 1,046) identified with more than one sexual orientation; queer was the most frequently endorsed exclusive sexual orientation. There were no differences across gender minority subgroups for education although a majority (66.3%, n = 1,221) of individuals had at least a 2-year college degree or higher. There were no differences across gender minority subgroups in proportions of people who screened positive across the different screening methods or in the alcohol use outcomes.

**Table 1:** Demographic characteristics of gender minority participants of The PRIDE study who completed alcohol measures (N = 1,892) by gender.

Participant Characteristics *	Gender-expansive (*n* = 1,239)	Transfeminine (*n* = 232)	Transmasculine (*n* = 421)	*P-Value*
**Age, mean, median (SD)**	29.83, 27.22 (9.78)	40.98, 38.04 (14.60)	30.91, 28.24 (10.39)	<.001
**Race/Ethnicity, n (%)**				.12 †
American Indian/Alaska Native	2 (0.16)	0 (0.00)	1 (0.24)	
Asian	40 (3.23)	2 (0.86)	7 (1.66)	
Black/African American	12 (0.97)	2 (0.86)	11 (2.61)	
Hispanic, Latino, or Spanish	14 (1.13)	3 (1.29)	7 (1.66)	
Middle Eastern or North African	4 (0.32)	1 (0.43)	1 (0.24)	
White	994 (80.23)	199 (85.78)	346 (82.19)	
Multiple Races and/or Ethnicities	162 (13.08)	18 (7.76)	48 (11.40)	
Another Race or Ethnicity	11 (0.89)	7 (3.02)	0 (0.00)	
**Sexual Orientation, n (%)**				<.001 †
Asexual	44 (3.58)	11 (4.82)	10 (2.44)	
Bisexual	66 (5.37)	20 (8.77)	37 (9.02)	
Gay	22 (1.79)	1 (0.44)	49 (11.95)	
Lesbian or Gay	59 (4.80)	53 (23.25%)	1 (0.24)	
Pansexual	47 (3.82)	28 (12.28)	23 (5.61)	
Queer	184 (14.97)	8 (3.51)	73 (17.80)	
Same-Gender Loving	1 (0.08)	1 (0.44)	0 (0.00	
Straight/Heterosexual	1 (0.08)	15 (6.58)	40 (9.76)	
More than 1 SO	787 (64.04)	85 (37.28)	174 (42.44)	
Questioning/Other	18 (1.46)	6 (2.63)	3 (0.73)	
**Highest Level of Education Attained, n (%)**				.51
Less than high school completion	13 (1.08)	1 (0.44)	5 (1.22)	
High school diploma or equiv.	388 (32.23)	77 (33.77)	139 (33.82)	
College degree (2- or 4-year)	481 (39.95)	102 (44.74)	157 (38.20)	
Graduate degree	323 (26.83)	48 (21.05)	110 (26.76)	
**Income, n (%)**				<.001
<$20,000	630 (53.12)	91 (40.62)	204 (50.50)	
$20,001–60,000	411 (34.65)	67 (29.91)	140 (34.65)	
$60,001–100,000	96 (8.09)	40 (17.86)	45 (11.14)	
$100,001+	49 (4.13)	26 (11.61)	15 (3.71)	
**Positive screens by method, n (%)**				
How long has it been since you last had 4 or more drinks containing alcohol on one occasion?	614 (49.56)	105 (45.26)	193 (45.84)	.27
How long has it been since you last had 5 or more drinks containing alcohol on one occasion?	504 (40.68)	85 (36.64)	162 (38.48)	.44
How often do you have a drink containing alcohol? *(AUDIT Item 1*,≥ *2–4 times a month)*	645 (52.06)	119 (51.29)	202 (47.98)	.35
How many drinks containing alcohol do you have on a typical day when you are drinking? *(AUDIT Item 2*, ≥ *3–4 drinks)*	235 (18.97)	47 (20.26)	85 (20.19)	.81
AUDIT-C using cutoff score of 3	483 (38.98)	96 (41.38)	161 (38.24)	.72
AUDIT-C using cutoff score of 4	286 (23.08)	54 (23.28)	94 (22.33)	.94
**Positive for outcome, n (%)**				
Harmful alcohol use	169 (13.64)	30 (12.93)	44 (10.45)	.24
Alcohol dependence symptoms and consequences	421 (33.98)	64 (27.59)	124 (29.45)	.06

*All percentages calculated based on available participant data, not overall sample N

^†^Chi-square was calculated on collapsed categories for these items due to small expected cell counts and limitations of statistical software to calculate Fisher’s exact test for this quantity of cells

### Potential screening methods for harmful drinking

Among all gender minority people considered together and among all subgroups, “5 or more” drinks maximized sensitivity and specificity as a screening method for harmful drinking (AUC values as well as sensitivity and specificity for the screening methods can be found in Table 2). Among all gender minority people considered together, there was a difference in AUC values for “4 or more” drinks (0.79, 95% CI: 0.77–0.80) versus “5 or more” drinks (0.83, 95% CI: 0.81–0.84) in predicting harmful alcohol use (*χ*^2^[1] = 46.68, *p* < .001). Among gender-expansive people, there was a difference between the AUC value for “4 or more” drinks (0.78, 95% CI: 0.76–0.80) versus “5 or more” drinks (0.82, 95% CI: 0.80-.84; *χ*^2^[1] = 32.10, *p* < .001). Among transmasculine individuals and transfeminine individuals, it was not possible to compare AUC values using the *roccomp* procedure due to perfect prediction of the outcome (*i*.*e*., there were 0 transmasculine or transfeminine participants who had an AUDIT score ≥ 8 and denied drinking “4 or more” drinks in the past year). While statistical comparison of the AUC values was not possible, observed AUC values were greater for “5 or more” drinks for both transmasculine (0.83, 95% CI: 0.80–0.86) and transfeminine (0.86, 95% CI: 0.83–0.89) groups when compared to AUC values for “4 or more” drinks (0.80, 95% CI: 0.78–0.83; and 0.81, 95% CI: 0.78–0.85, respectively). There was no difference in the AUC values of “5 or more” drinks between gender-expansive and transmasculine individuals. Comparison of the AUC value of “5 or more” drinks for transfeminine individuals to the AUC values for the other two gender minority subgroups was not possible due to perfect prediction among transfeminine individuals (*i*.*e*., no transfeminine individuals had an AUDIT score ≥ 8 and denied drinking “5 or more” drinks in the past year).

**Table 2:** Sensitivity (sens), specificity (spec), area under the curve (AUC), positive predictive value (PPV), and negative predictive value (NPV) of screening methods for harmful alcohol use and alcohol dependence symptoms and consequences among gender minority participants of The PRIDE study (N = 1892).

Screening method	All gender minority people					Gender-expansive					Transfeminine					Transmasculine				
	Sens (%)	Spec (%)	AUC (95% CI)	PPV (%)	NPV (%)	Sens (%)	Spec (%)	AUC (95% CI)	PPV (%)	NPV (%)	Sens (%)	Spec (%)	AUC (95% CI)	PPV (%)	NPV (%)	Sens (%)	Spec (%)	AUC (95% CI)	PPV (%)	NPV (%)
Harmful alcohol use*																				
4 drinks/occasion	98.77	59.25	0.79 (0.77–0.80)	26.32	99.69	98.22	58.13	0.78 (0.76–0.80)	27.04	99.52	100.00	62.87	0.81 (0.78–0.85)	28.57	100.00	100.00	60.48	0.80 (0.78–0.83)	22.80	100.00
5 drinks/occasion	97.12	68.77	0.83 (0.81–0.84)	31.42	99.39	96.45	68.13	0.82 (0.80–0.84)	32.34	99.18	100.00	72.77	0.86 (0.83–0.89)	35.29	100.00	97.73	68.44	0.83 (0.80–0.86)	26.54	99.61
**Alcohol dependence symptoms & consequences**†																				
4 drinks/occasion	86.37	69.91	0.78 (0.76–0.80)	57.68	91.53	85.99	69.19	0.78 (0.75–0.80)	58.96	90.56	89.06	71.43	0.80 (0.75–0.85)	54.29	94.49	86.29	71.04	0.79 (0.75–0.83)	55.44	92.54
5 drinks/occasion	76.68	77.86	0.77 (0.75–0.79)	62.18	87.55	76.48	77.75	0.77 (0.75–0.80)	63.89	86.53	82.81	80.95	0.82 (0.76–0.87)	62.35	92.52	74.19	76.43	0.75 (0.71–0.80)	56.79	87.64
AUDIT1: Frequency of drinking	83.42	64.30	0.74 (0.72–0.76)	52.59	89.09	82.42	63.57	0.73 (0.71–0.75)	53.80	87.54	85.94	61.90	0.74 (0.68–0.80)	46.22	92.04	85.48	67.68	0.77 (0.72–0.81)	52.48	91.78
AUDIT2: Number of drinks on a typical day	42.36	91.50	0.67 (0.65–0.69)	70.30	76.98	40.86	92.30	0.67 (0.64–0.69)	73.19	75.20	45.31	89.29	0.67 (0.61–0.74)	61.70	81.08	45.97	90.57	0.68 (0.64–0.73)	67.06	80.06
AUDIT-C, cutoff score 3 or more	75.53	78.18	0.77 (0.75–0.79)	62.16	87.07	75.06	79.58	0.77 (0.75–0.80)	65.42	86.11	82.81	74.40	0.79 (0.73–0.84)	55.21	91.91	73.39	76.43	0.75 (0.70–0.80)	56.52	87.31
AUDIT-C, cutoff score 4 or more	51.72	90.72	0.71 (0.69–0.73)	72.58	79.84	50.36	90.95	0.71 (0.68–0.73)	74.13	78.07	57.81	89.88	0.74 (0.67–0.80)	68.52	84.83	53.23	90.57	0.72 (0.67–0.77)	70.21	82.26

*Harmful alcohol use was measured on the AUDIT using a cut point of ≥8

^†^Alcohol dependence symptoms and consequences were the endorsement of ≥ 1 experiences or symptoms on the AUDIT items 4–9

### Potential screening methods for alcohol dependence symptoms and consequences

For AUDIT items 1 and 2, cutoff values were determined first so that these screening methods could be compared to the other candidate screening methods. AUDIT item 1 (“how often did you have a drink…”) was optimized in predicting one or more alcohol dependence symptoms or consequences with the greatest AUC values at a score of 2 or more, corresponding to having a drink containing alcohol 2–4 times a month or more for all groups. AUDIT item 2 (“how many drinks …did you have on a typical day…”) was optimized in predicting one or more alcohol dependence symptoms or consequences with the greatest AUC values at a score of 1 or more for all groups, corresponding to having 3 or 4 drinks on a typical day. These cut-points were then used in subsequent analyses comparing the potential screening measures.

Among all gender minority people considered together, there was a difference between the AUC values of the screening methods in predicting one or more alcohol dependence symptoms or consequences (*χ*^2^[5] = 114.63, *p* < .001). Using “4 or more” drinks, “5 or more” drinks, and the AUDIT-C with a cutoff score of 3 points all had the highest AUC values (0.78, 0.78, and 0.77, respectively) and were not detectably different from one another (*χ*^2^[2] = 2.07, *p* = .36).

Among gender-expansive people, there was a difference in screening method performance in predicting one or more alcohol dependence symptoms or consequences (*χ*^2^[5] = 89.81, *p* < .001). Among gender-expansive people, the screening methods of “4 or more” drinks (0.78), “5 or more” drinks (0.77), and the AUDIT-C with a cutoff score of 3 points (0.77) had the highest AUC values with no detectable differences between these screening measures (*χ*^2^[2] = 0.24, *p* = .89).

Among transmasculine people, there was a difference in the AUC values for models predicting one or more alcohol dependence symptoms or consequences (*χ*^2^[5] = 16.62, *p* = .005). The screening method of “4 or more” drinks (0.79), AUDIT item 1 (0.77), “5 or more” drinks, and the AUDIT-C cutoff score of 3 had the highest AUC values (0.75) with no detectable difference between the AUC values for any of these screening methods (*χ*^2^[3] = 4.95, *p* = .18).

Among transfeminine people, there was a difference in AUC values for models predicting one or more alcohol dependence symptoms or consequences (*χ*^2^[5] = 23.22, *p* < .001). The screening method of “5 or more” drinks (0.82), “4 or more” drinks (0.80), and the AUDIT-C cutoff score of 3 (0.79) had the highest AUC values with no detectable difference between the AUC values of these methods (*χ*^2^[2] = 1.39, *p* = .50).

### Comparing screening measures between gender minority subgroups

The methods with the greatest AUC values were compared to evaluate differences between each of the gender minority subgroups. There were no differences in the AUC values of “4 or more” drinks (*χ*^2^[2] = 0.93, *p* = .63), “5 or more” drinks (*χ*^2^[2] = 3.38, *p* = .18), AUDIT-C cutoff score of 3 (*χ*^2^[2]] = 1.16, *p* = .56), or the AUDIT 1 item (which was only among the highest AUC for transmasculine individuals; *χ*^2^[2] = 2.16, *p* = .34) between gender minority subgroups.

## Discussion

This study sought to provide guidance regarding which brief screening measures should be used in screening for harmful drinking and alcohol dependence symptoms and consequences with gender minority people generally and among gender minority subgroups. Our results showed that screening for “5 or more” drinks on one occasion in the past year among gender minority people performed better than screening for “4 or more” drinks when screening for harmful drinking as measured by the full ten-item AUDIT. Screening for “5 or more” drinks maximized sensitivity and specificity in screening for harmful alcohol use when all gender minority people were considered together and when gender-expansive, transmasculine, and transfeminine people were considered separately. Screening using “5 or more” drinks on one occasion had greater specificity (range: 68%-73%) than “4 or more” drinks (range: 58%-63%) while maintaining high levels of sensitivity among all groups (range: 96%-100%).

When we examined screening methods for the occurrence of one or more alcohol dependence symptoms or consequences, as defined by items 4–9 on the AUDIT [16] and not dependence symptoms derived from diagnostic criteria [21], the screening methods of “4 or more” drinks on one occasion, “5 or more” drinks on one occasion, and the AUDIT-C (using the cutoff score of 3 or more) all maximized sensitivity and specificity and were not detectably different from each other. This suggests that any of these methods could be used to screen for alcohol dependence symptoms and consequences in this population. These screening measures maximized sensitivity and specificity among all gender minority people considered together and among gender-expansive, transmasculine, and transfeminine people considered separately. Other measures considered (*i*.*e*., AUDIT Item 1, AUDIT Item 2, and the AUDIT-C cutoff score of 4) did not perform as well in screening for alcohol dependence symptoms and consequences in this population, except for among transmasculine individuals where AUDIT Item 1, which queries frequency of alcohol use, performed equally well. The AUC values of these screening measures did not differ between gender minority subgroups, suggesting similar maximization of sensitivity and specificity between groups.

While the results of our study suggest that multiple measures could be used, screening for “5 or more” drinks on one occasion maximized sensitivity and specificity for all gender minority subgroups in predicting *both* harmful drinking *and* one or more alcohol dependence symptoms or consequences. The screening method of “5 or more” drinks on one occasion was derived as a gender-based guideline to be used for “men,” without guidance to what “men” meant within this context and with a presumption that everyone is cisgender. We recommend that clinicians use “5 or more” drinks on one occasion within the past year as a screening measure for gender minority people until such a time that a new, more robust screening measure is developed and validated within this population. While other potential screening candidates (*i*.*e*., “4 or more” drinks on one occasion or the AUDIT-C with a cutoff score of 3) performed well in each gender minority subgroup to predict alcohol dependence and consequences, none of these screening methods performed better than screening with “5 or more” drinks. Furthermore, “5 or more” drinks on one occasion is a single item that can be administered more easily than the 3-item AUDIT-C, which must be both administered and scored. As there was no difference in performance and “5 or more” drinks on one occasion maximized sensitivity and specificity for *both* clinical outcomes tested here, we recommend using this screening method in clinical settings. This screening recommendation provides easy and clear guidance for health care professionals trying to determine best practice screening methods for harmful alcohol use among gender minority people.

### Limitations

Rather than utilizing diagnostic criteria as the outcome to identify screening measures, we chose harmful alcohol use and the existence of one or more AUDIT-derived alcohol dependence symptoms or consequences. We chose the AUDIT because it is highly related to robustly assessed clinical outcomes [22] and because more extensive diagnostic screening was not possible with this sample. It is important to note that alcohol dependence symptoms and consequences, as defined here, do not map onto current diagnostic criteria [21], and instead reflect a subset of the AUDIT, similar to a method used by Bradley et al. to establish alcohol screening methods in a different population [18]. While we considered only examining harmful alcohol use as an outcome in this study, we determined that using two outcomes (*i*.*e*., harmful alcohol use *and* alcohol dependence symptoms or consequences) would allow us to determine if different screening methods were more applicable to different alcohol related outcomes. In this study, our results suggest that a single screening method: “5 or more” drinks performed well in predicting both of our outcomes, strengthening our recommendation to use this as a screening method for this population. This study also considered only limited alcohol screening measures. Future studies may consider additional screening measures (*e*.*g*., CAGE, Short Michigan Alcohol Screening Test) and may find that these other measures outperform the measures selected here. This study was not conducted in a clinical setting. Direct entry of answers by participants, as is done in The PRIDE Study, may have increased willingness to report alcohol use [23]; these screening measures may perform differently when administered by a health care provider or in a clinical setting. The PRIDE Study is comprised of people who volunteer to participate and does not represent a probabilistic sample of persons within the United States; the results reported here may lack external validity and should be replicated in other samples. This sample was also predominantly White; thus, this study should be replicated in samples with greater representation of other ethno-racial groups. In this study, we used the AUC as the criteria to compare measures. This approach assumes equal weight on the importance of sensitivity and specificity. In some clinical contexts, one may be more important than the other, and departure from the screening method that we recommend may be warranted. Furthermore, in this study, we did not adjust for multiple comparisons; some of the detected differences between measures may be spurious. We made this decision to equally weigh the cons of a Type II error (*i*.*e*., not detecting a difference that was there) and a Type I error (*i*.*e*., detecting a difference falsely) in making clinical recommendations for this population [24]. Despite this concern, given the pattern of results observed here, we expect that our overall recommendations are not spurious.

## Conclusions

In sum, our study found that screening for “‘5 or more’ drinks on one occasion within the past year” performed better than or as well as other measures in detecting harmful drinking and one or more alcohol dependence symptoms or consequences among gender minority people. Within the current landscape of gender-based screening guidelines for harmful drinking and limited information on how screen gender minority people, we recommend using a single item to query if the person has had “5 or more” drinks on one occasion within the past year to identify if further alcohol assessment and intervention is recommended among gender minority people.

## Supporting information

S1 Table Definitions relevant to gender, gender minority people, and sexual minority people. (DOCX)
