# Supplementary material for: Screening gender minority people for harmful alcohol use
Source: PLoS One. 2020 Apr 7;15(4):e0231022. doi: 10.1371/journal.pone.0231022 (PMC7138294; doi:10.1371/journal.pone.0231022)
Supplement: S1 Table — (DOCX) [file pone.0231022.s001.docx]

S1 Table. Definitions relevant to gender, gender minority people, and sexual minority people

| Cisgender | A term typically used in research and academic settings to describe people who have a gender or gender identity that aligns with what is commonly associated with the sex assigned to them at birth (*i.e..,* woman and female; man and male). |
| --- | --- |
| Gender | A social construct that can include, at minimum, an internal understanding of one’s internal gender, gender expression, and gender identity. |
| Gender expansive | A term to describe people who have a gender that does not align with a feminine or masculine binary. This may include people who are non-binary, genderqueer, or who identify with more than one binary gender. |
| Gender expression | They way in which someone outwardly expresses their gender. |
| Gender identity | The gender with which someone identifies (*e.g.,* agender, genderqueer, man, non-binary, transfeminine, transman, transmasculine, transwoman, woman). This can only be known by asking someone. Gender identity labels are rapidly changing, particularly among people who identify as a gender minority person. |
| Gender minority | A term typically used in research and academic settings to describe people who have a gender or gender identity that does not exclusively align with what is commonly associated with the sex assigned to them at birth (*e.g.,* gender non-binary and female, woman and male). |
| LGBTQ+ | A term used to describe people who are lesbian, gay, bisexual, transgender, queer, questioning, or have another gender identity and/or sexual orientation that is not exclusively cisgender *and* heterosexual/straight. LGBTQ+ is often used synonymously with the term sexual and/or gender minority (SGM). |
| Sex assigned at birth | The sex (*e.g.,* male, female,) that was assigned to a person at birth, typically by reviewing genitalia but sometimes through medical imaging or genetic analysis and commonly documented on one’s birth certificate. |
| Sexual minority | A term typically used in research and academic settings to describe people who have a sexual orientation that is not exclusively heterosexual. |
| Sexual orientation | Sexual orientation is comprised of, at minimum, sexual attraction, sexual behavior, and identity (*e.g.,* bisexual, gay, heterosexual, lesbian, pansexual, queer, straight). Like gender identity labels, sexual orientation identity labels are rapidly changing, particularly among people who identify as a sexual minority person. |
| Transfeminine | A term used to describe people who are transgender and on a feminine spectrum (*e.g.,* identify as girl, transwoman, woman). |
| Transgender | A person who has a gender that does not align with what is commonly associated with the sex assigned to them at birth. Transgender is used frequently to refer to people on a binary gender spectrum (*e.g.,* transgender woman, transgender man, man, woman) but definitions may at times also include people who have a gender expansive identity. |
| Transmasculine | A term used to describe people who are transgender and on a masculine spectrum (*e.g.,* identify as boy, man, transman). |
